# Analysis of Mitochondrial Function, Structure, and Intracellular Organization In Situ in Cardiomyocytes and Skeletal Muscles

**DOI:** 10.3390/ijms23042252

**Published:** 2022-02-18

**Authors:** Andrey V. Kuznetsov, Sabzali Javadov, Raimund Margreiter, Judith Hagenbuchner, Michael J. Ausserlechner

**Affiliations:** 1 Cardiac Surgery Research Laboratory, Department of Cardiac Surgery, Innsbruck Medical University, A-6020 Innsbruck, Austria; 2 Department of Pediatrics I, Medical University of Innsbruck, A-6020 Innsbruck, Austria; 3 Department of Physiology, School of Medicine, University of Puerto Rico, San Juan, PR 00936-5067, USA; sabzali.javadov@upr.edu; 4 Department of Transplant Surgery, Medical University of Innsbruck, A-6020 Innsbruck, Austria; raimund.margreiter@tirol-kliniken.at; 5 Department of Pediatrics II, Medical University of Innsbruck, A-6020 Innsbruck, Austria; judith.hagenbuchner@i-med.ac.at

**Keywords:** cardiac mitochondria, mitochondrial respiratory function, confocal fluorescent imaging, mitochondrial intracellular organization, heterogeneity, oxidative phosphorylation, reactive oxygen species, mitochondrial swelling

## Abstract

Analysis of the function, structure, and intracellular organization of mitochondria is important for elucidating energy metabolism and intracellular energy transfer. In addition, basic and clinically oriented studies that investigate organ/tissue/cell dysfunction in various human diseases, including myopathies, cardiac/brain ischemia-reperfusion injuries, neurodegenerative diseases, cancer, and aging, require precise estimation of mitochondrial function. It should be noted that the main metabolic and functional characteristics of mitochondria obtained in situ (in permeabilized cells and tissue samples) and in vitro (in isolated organelles) are quite different, thereby compromising interpretations of experimental and clinical data. These differences are explained by the existence of the mitochondrial network, which possesses multiple interactions between the cytoplasm and other subcellular organelles. Metabolic and functional crosstalk between mitochondria and extra-mitochondrial cellular environments plays a crucial role in the regulation of mitochondrial metabolism and physiology. Therefore, it is important to analyze mitochondria in vivo or in situ without their isolation from the natural cellular environment. This review summarizes previous studies and discusses existing approaches and methods for the analysis of mitochondrial function, structure, and intracellular organization in situ.

## 1. Introduction

Mitochondria are involved in numerous metabolic pathways [1,2,3,4,5,6,7] and produce a major portion of intracellular ATP through the electron transport chain (ETC) in some cells, coupled with oxidative phosphorylation; they provide over 90% of cellular ATP in high oxygen consuming organs such as the heart, liver, and brain. Mitochondria are composed of two separate and functionally different membranes, the outer mitochondrial membrane (OMM) and the inner mitochondrial membrane (IMM), with the intermembrane space between them and the internal space known as the matrix enclosed by the IMM [6]. These organelles contain the circular genome, mitochondrial DNA (mtDNA), which was reduced in size during evolution through deletions or point mutations and its transfer to the nucleus. The size, shape, and number of mitochondria vary in different cell types. Cells contain independent and/or interconnected mitochondria, which create the subcellular mitochondrial network. In addition to ATP production, mitochondria also participate in other aspects of cell metabolism and physiology, such as the regulation of ion homeostasis, particularly intracellular Ca^2+^, fatty acid and cholesterol metabolism, redox status, cell survival, and cell death mechanisms [2,4,8,9,10,11,12,13,14]. Mitochondria bioenergetics and intracellular energy transfer are involved in the pathogenesis of numerous metabolic and hereditary diseases, including cardiovascular and neurodegenerative diseases, diabetes, cancer, and aging [15,16,17,18,19,20,21,22,23,24,25,26,27]. Mitochondria are the major source of reactive oxygen species (ROS) that, at low concentrations, participate in cellular signalling mechanisms whereas, at high concentrations, cause oxidative stress and cell death [22,23,24,25,26,27,28]. Notably, energy metabolism and crosstalk between cytoplasmic and mitochondrial ATP production are markedly different in cancer cells compared to non-cancer cells in terms of adaptation to environmental conditions. Therefore, a multifaceted and comprehensive analysis of mitochondrial function and morphology is important for the precise estimation of cell physiology and pathophysiology. In the following sections, we will discuss the biochemical and biophysical approaches that are currently applied to analyze the function and structural organization of mitochondria in situ or in vivo using techniques that do not require isolation of the organelles.

## 2. Advantages of Using Intact Cells or In Situ Approaches for Mitochondria Research

**Figure 1:**
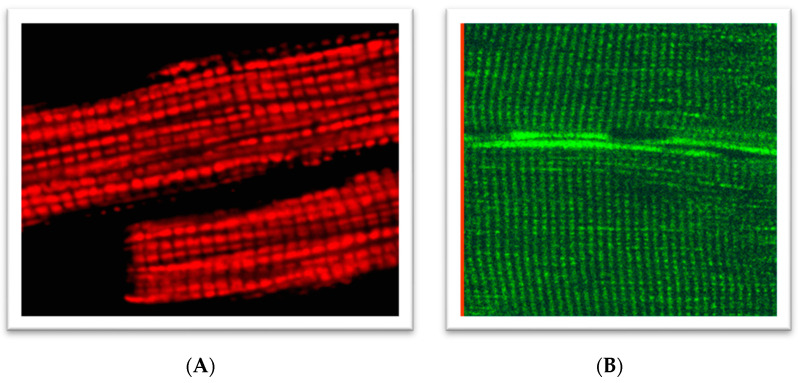
Regular arrangements of mitochondria in: (**A**) rat cardiomyocytes loaded with 0.1 µM tetramethylrhodamine methyl ester (TMRM), 30 min incubation of cells at room temperature (543 nm laser excitation); (**B**) rat skeletal (quadriceps) muscles (flavoproteins autofluorescence, 488 nm laser excitation). A portion of the figure was reprinted with permission from ref [38,39]. Copyright 2006 Elsevier.

Mitochondrial oxygen consumption rates can be determined using a traditional Clark electrode in isolated mitochondria [29,30,31,32,33] in vitro and non-isolated mitochondria of intact permeabilized cells or muscle fibers in situ [6,34,35,36,37]). In addition, cytometry, capillary electrophoresis, patch-clamping, and optical trapping were used for the analysis of mitochondria [32]. Analysis of mitochondria in muscle biopsies remains an important initial screening procedure for the potential presence of metabolic diseases in humans. The changes in the mitochondrial structure, dynamics, organization, interaction with other cell systems, and several functional properties, such as redox state, mitochondrial ROS (mtROS), biogenesis, etc., can be analyzed with a confocal fluorescent imaging approach using TMRM or flavoproteins fluorescence (Figure 1) in situ or in vivo [6,38,39,40].

**Figure 2:**
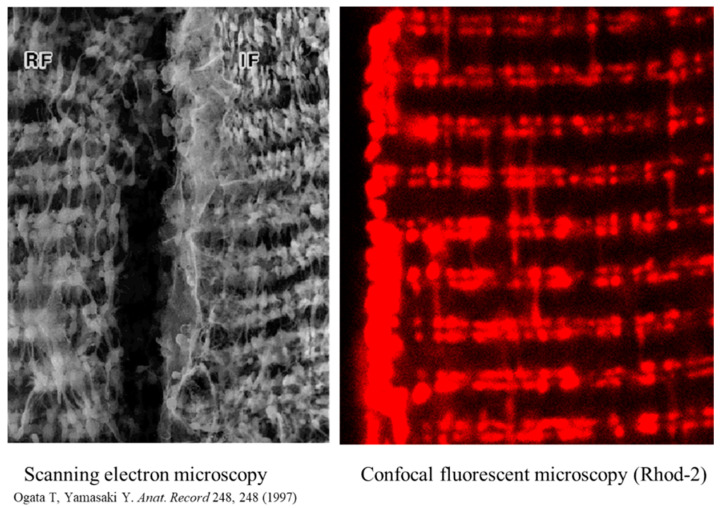
Mitochondrial intracellular arrangement in skeletal muscles, visualized by ultra-high-resolution scanning electron microscopy and in muscles loaded (60 min, room temperature) with 5 µM of the specific fluorescent mitochondrial Ca^2+^ probe Rhod-2AM. Reprinted with permission from ref [41,42]. Copyright 2006 Elsevier. Rhod-2 has a net positive charge, thereby allowing for its specific accumulation in mitochondria. Reprinted with permission from ref [41]. Copyright 2006 Elsevier. Fluorescence was measured using 543 nm for excitation (helium-neon laser) and greater than 580 nm for emission. Note: A portion of the figure was reprinted with permission from ref [39,43]. Copyright 2006 Elsevier.

Figure 2 compares imaging of mitochondria in skeletal muscles performed by ultra-high-resolution scanning electron microscopy [41] to the specific fluorescent mitochondrial Ca^2+^ probe Rhod-2 [39]. These two images bear several similarities in terms of mitochondrial shapes, intracellular positions, and organization of these organelles in muscles, including numerous inter-mitochondrial contacts and clusters of the subsarcolemmal mitochondrial subpopulation. The major difference is that for electron microscopy, muscles were always fixed. In contrast, muscles for fluorescent imaging were not fixed and mitochondria were functional, thereby preserving their capacity for dynamics and accessibility for various experimental manipulations.

A specific protocol was developed for the analysis of functional mitochondria in situ, without isolation of organelles, in selectively permeabilized cells or muscle fibers using digitonin or saponin, which only permeabilize the plasma membrane, thereby leaving mitochondrial membranes intact [6,33,34,35,36]. Digitonin and saponin specifically interact and solubilize cholesterol in the plasma membrane and thus permeabilize the membrane, leading to the formation of non-selective pores. The plasma membrane contains high cholesterol amounts, whereas the membranes of the sarco/endoplasmic reticulum or mitochondria have considerably lower cholesterol contents. Based on these differences, chemical permeabilizing agents, such as saponin, digitonin, filipin, solanine, tomatine, or alamethicin, selectively permeabilize the cells or muscle fibers [6,44,45,46,47,48,49,50,51], leaving the intracellular membranes of the mitochondria, sarco/endoplasmic reticulum, myofilaments, and cytoskeleton intact, and equilibrating the intracellular spaces with incubation medium. Therefore, the cell permeabilization technique provides a unique possibility to analyze human biopsies or genetically modified animals and can be used in basic research as well as for the diagnosis of various human diseases in clinically oriented studies.

Enzymatic assays of individual respiratory (ETC) complexes or other mitochondrial enzymes are widely used for the estimation of mitochondrial function under physiological and pathological conditions [52,53,54]. However, this method has been shown to be insufficient for the accomplished analysis of mitochondrial function and injury since all enzymes and their complexes interact with each other. Hence, oxidative phosphorylation should be investigated in intact mitochondria [32,55,56] through the measurement of oxygen consumption [57,58]. Standard procedures of organelle isolation based on differential centrifugation of tissue or cell homogenates allow for a precise characterization of functional properties of mitochondria in vitro [32]. Although remarkable knowledge has been gained using isolated mitochondria, this technique has several serious limitations: (i) it requires relatively large amounts of tissues or cells; (ii) mitochondria are frequently damaged by the isolation (several centrifugations and washings) procedure [59,60]; and (iii) mitochondria isolated from cells and tissues lack their essential intracellular environment. The isolation procedure has been shown to result in changes to mitochondrial morphology, sensitization to permeability transition pore (PTP) opening, changes in respiratory mitochondrial function, and increased mitochondrial ROS production [6,59,60]. Moreover, mitochondrial networks and all contacts of mitochondria within the sarco/endoplasmic reticulum and cytoskeleton are disrupted during isolation, which significantly affects their structural organization, metabolism, and physiology.

Cells are highly organized units with multiple and multifaceted functional and structural interactions between various subcellular systems. A large number of studies provide strong evidence that elucidating individual organelles alone is not sufficient, and systemic approaches must be applied for a better understanding of cell physiology, crosstalk between organelles, and cellular signaling pathways. Since mitochondria actively interact with the cytoskeleton and sarco/endoplasmic reticulum [8,12,61,62,63,64,65], the interaction between mitochondria and cytoskeletal proteins (plectin and tubulin beta II) and their connections beyond the voltage-dependent anion channel (VDAC) can be studied only in vivo or in situ. These interconnections are ultimately involved in the regulation of mitochondrial function and can be studied using combinations of several modern techniques. Several cytoskeletal elements play a vital role in the structural and functional organization of mitochondria, including mitochondrial morphology, dynamics, motility, and mitosis. In the heart, mitochondrial bioenergetics and oxygen consumption are linearly dependent on the cardiac contractile activity [66,67] and rather stable concentration of ADP, a regulator of mitochondrial respiration. The apparent Michaelis constant (appKm) for ADP is an important parameter for mitochondrial respiration; it indicates the affinity of mitochondrial respiration to ADP (response to ADP), thereby reflecting the permeability state of the OMM [63,68] that is calculated from the respiratory ADP kinetics. A high response to ADP (low appKm, about 10–30 µM) has been obtained for isolated mitochondria in vitro [58,59]. Interestingly, measurements of mitochondrial respiration in situ (e.g., in permeabilized cells) showed more than 10-fold higher (200–350 µM) appKm ADP values, demonstrating that their interaction with cytoskeletal proteins (plectin, tubulin beta II) could be essential for the permeability of the OMM and, therefore, for the regulation of mitochondrial respiratory function since the absence of certain cytoskeletal proteins leads to low appKmADP [69,70]. To overcome the limitations associated with isolated mitochondria and simultaneously maintain a similar high scope for experimental manipulations, a new method for studying the function of intact or permeabilized cells, muscle fibers, or tissue homogenates has been established [6,34,35,36,37,44,45,46,47,48,49,50,51]. This approach uses the capacity of biological detergents (mostly digitonin and saponin) to specifically interact with the plasma membrane cholesterol of cells or muscle fibers. In permeabilized cells or muscle fibers, the organization, structure, and function of mitochondria and the cytoskeletal proteins remain mostly intact [35,36,37]. The respiratory control ratio (state 3, after ADP addition)/(state 2, before ADP addition) in permeabilized muscle (e.g., quadriceps muscles) fibers can be even higher than in carefully isolated mitochondria. Moreover, mitochondria become fully accessible to specific substrates or inhibitors in these preparations and can be used for the mitochondrial respiratory analysis while remaining connected to other cellular systems. This is especially important considering recent evidence of enzymatic/metabolic channeling enzyme redistribution and nucleotides (ADP/ATP) compartmentation in the cell [63,71,72].

## 3. Analysis of ETC Complexes

A specifically designed substrate/inhibitor/uncoupler titration protocol is applied for a step-by-step functional analysis of mitochondrial ETC complexes (I, II, III, IV) under in situ-like conditions. Notably, numerous studies using permeabilized muscle fibers or cells confirmed that mitochondria are capable of utilizing various substrates (e.g., glutamate, malate, pyruvate, succinate, TMPD/ascorbate) with a high sensitivity to specific inhibitors of mitochondrial ETC complexes (rotenone, antimycin A, etc.), thereby demonstrating functional intactness of well-coupled mitochondria [6,73,74,75]. In addition, the analysis of β-oxidation of fatty acids or glutamine oxidation, medium- and long-chain acylcarnitines may also be used for the measurements [6,76]. The uncoupled maximum respiration flux can be induced by the addition of frequently used uncouplers such as CCCP or FCCP to collapse the proton-motive force through the IMM and thus estimate the maximum mitochondrial capacity. Since CCCP and FCCP are membrane-permeable agents, they can be used in intact living cells without permeabilization to estimate cellular respiration under uncoupled conditions and maximal mitochondrial capacity. CCCP and FCCP must be used at optimal concentrations since they have inhibitory effects at extremely high concentrations. Usually, the uncoupled control ratio (UCR) [73,74,75,77] in intact cells is in a range of 4.3–4.5. Importantly, endogenous and uncoupled respirations were found to be linearly dependent on the cell density in a range of 0.2–6.0 × 10^6^ cells/mL. This mitochondrial uncoupling has also been suggested for a cardioprotection strategy under oxidative stress, diabetes, and ischemia-reperfusion injury [77,78]. In addition to respiratory parameters, analysis of the mitochondrial membrane potential using different fluorescent potential-dependent dyes (e.g., TMRM, JC-1) by FACS analysis and other techniques provides useful additional information about mitochondrial function.

## 4. Cytochrome *c* Test for the Assessment of the Intactness of OMM

Cytochrome *c* is a soluble peripheral membrane protein localized in the intermembrane space, which is only loosely bound to the IMM; this membrane facilitates the transport of electrons to complex IV, whereas other cytochromes represent integral proteins. When the OMM is intact, cytochrome *c* remains in the intermembrane space and the addition of exogenous cytochrome *c* does not affect respiration. However, if the OMM is damaged, the endogenous cytochrome *c* can be released from the IMS at physiological ionic strength. In this case, the exogenous cytochrome *c* added to the experimental chamber will remarkably stimulate the respiratory rates in permeabilized cells and muscle fibers [77]. After the addition of a saturating concentration of cytochrome *c*, the maximal respiration downregulated by cytochrome *c* depletion can be restored. In particular, the cytochrome *c* test is recommended to be performed in cases of multiple respiratory chain defects. The level of cytochrome *c* in mitochondria and its release into the cytosol is estimated in studies that elucidate the role of mitochondria in apoptosis [79].

## 5. Mitochondrial Creatine Kinase Coupling and Energy Transfer

Several isoforms of creatine kinases (CKs) are involved in cell energy metabolism and intracellular energy transport, in particular, for the synthesis of phosphocreatine in the intermembrane space by means of the mitochondrial isoform of CK (mi-CK). In the heart and oxidative muscles, mi-CK is coupled with oxidative phosphorylation through the ATP-ADP carrier under normal conditions [63,71,72]. Therefore, the addition of creatine (creatine test) substantially increases mitochondrial respiration since mi-CK acts as an ADP-regenerating system. Similarly, mitochondrial AMP kinase or hexokinase can be the regenerating systems for ADP upon the addition of AMP or glucose [63,80]. Previous studies showed that coupling of mi-CK is significantly hampered in cardiac pathologies, such as ischemia-reperfusion injury or congestive heart failure [81]. Moreover, the mi-CK system is highly sensitive to oxidative stress due to the oxidation of essential SH groups [82]. Therefore, the mi-CK functional activity and coupling can be considered a sensitive parameter for evidencing alterations in mitochondrial physiology and cell bioenergetics and can be used as a diagnostic tool in cardiac injuries. The simple creatine test allows for the rapid evaluation of the functional (coupling) state of this mitochondrial enzyme in tissue biopsies. This test can be even more sensitive for the evaluation of the quality of permeabilized muscle fibers or cardiomyocyte preparations than simple measurements of fluxes.

More recently, a microplate-based assay (Seahorse assay [83]) of oxygen consumption rates was used for the assessment of cellular respiration (kinetics of oxygen concentration) in intact cells. In particular, this approach was successively applied to the measurement of respiration of non-permeabilized murine skeletal muscle cells [84,85,86,87]. The muscles were not permeabilized or mechanically dissected and were enzymatically dissociated to minimize manipulations that may affect the sample intactness prior to these measurements. The technologies allow for the measurement of cellular oxygen consumption in muscle fibers and various permeabilized or intact cells/tissues, thus estimating the cellular energy and ions (e.g., protons) in cells with intact physiology.

It should be noted that mitochondria in vivo are morphologically and functionally heterogeneous in cells [88,89], and this variety is thought to have important physiological consequences. It has been suggested that different mitochondrial subsets may perform diverse cellular functions depending on cellular demands [87]. Individual mitochondria may have different membrane potentials, mitoCa^2+^, mtROS, sensitivities to mitochondrial PTP induction, and even different motilities [90,91,92]. However, the precise mechanisms underlying the development of mitochondrial heterogeneity remain unknown.

## 6. Mitochondrial Swelling and Calcium Retention Capacity

Changes in the matrix volume induced by ions, particularly Ca^2+^ and K^+^, play a crucial role in the regulation of mitochondrial function and metabolism [93,94]. Modest increases in the matrix volume stimulate mitochondrial bioenergetics and ATP production [95,96], whereas excessive swelling of mitochondria due to sustained PTP opening impairs mitochondrial function, leading to cell death [97,98]. Mitochondrial Ca^2+^ overload accompanied by high mtROS levels is the main event that provokes PTP induction [99]. Due to the crucial role of mitochondria in human diseases [100], analysis of mitochondrial swelling is important for the estimation of mitochondrial damage induced by various pathological stimuli [101]. The Ca^2+^ retention capacity (CRC) represents the capability of mitochondria to uptake maximum Ca^2+^ and, therefore, is used to quantify the extent of PTP opening. Usually, the CRC is quantified in isolated mitochondria spectrophotometrically (light scattering) or using different Ca^2+^-sensitive dyes. For example, along with Ca^2+^-sensitive fluorescent dyes, arsenazo III, a non-membrane-permeant Ca^2+^-sensitive dye [101,102,103,104], and Ca^2+^-sensitive electrodes [104,105] have been used to measure Ca^2+^ release/PTP opening in isolated mitochondria. Notably, the estimation of the CRC/PTP opening in isolated mitochondria has several disadvantages [6,49,50].

The quantification of mitochondrial PTP/CRC in situ or in vivo was performed previously in cells and tissue samples. For example, mitochondrial PTP opening in the heart in vivo was quantified by the [^3^ H] 2-deoxyglucose (^3^ H-DOG) entrapment technique without isolation of mitochondria [106,107,108]. The isolated heart is perfused with ^3^ H-DOG, which is converted to ^3^ H-DOG-6-phosphate in the cytoplasm and enters the mitochondria through the PTP. The amount of ^3^ H-DOG-6-phosphate entrapped in mitochondria corresponds to the extent of PTP opening [109,110,111]. Calcein-AM, a cell-permeant fluorescent probe, is utilized to measure PTP/CRC in cells in situ, where Ca^2+^ release from mitochondria through the PTP indicates the extent of pore opening [112,113,114]. The technique for the measurement of PTP opening/CRC has certain disadvantages. In this approach, the fluorescence intensity of calcein in the cytosol is quenched by Co^2+^, a heavy metal, which exerts toxic effects on cells. Moreover, it is difficult to conclude whether calcein quenching is associated with its release through PTPs, or whether Co^2+^ enters through the PTP and quenches calcein in the matrix of mitochondria. In favor of this, cytosolic calcein has been shown to exit from normal mitochondria and enter back after PTP opening [115]. It should also be noted that calcein-AM is not cleaved in all cell types, such as hepatocytes [116].

**Figure 3:**
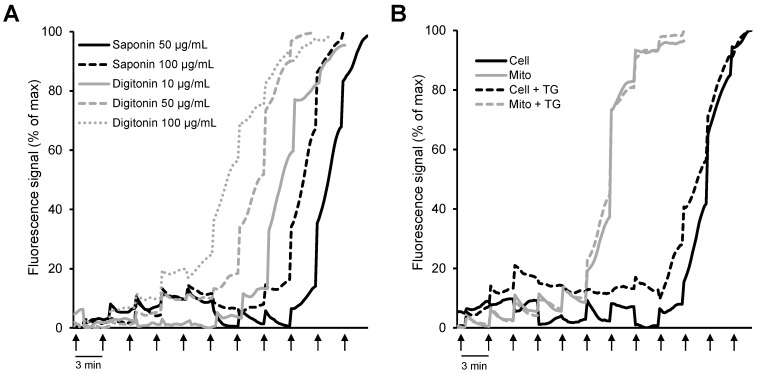
Comparative analysis of mitochondrial CRC in isolated mitochondria in vitro and permeabilized cells in situ: (**A**) H9c2 cardiomyocytes permeabilized by saponin (50 and 100 μg/mL) or digitonin (10, 50, and 100 μg/mL); (**B**) permeabilized H9c2 cardiomyocytes vs. isolated mitochondria in the presence or absence of 1 μM thapsigargin (TG, a SERCA inhibitor). Ca^2+^ was added every 3 min (*arrows*) by increments of 1 nmol/injection. (Note: the figure was reprinted with permission from ref [118]. Copyright 2006 Elsevier.)

In addition to mitochondrial bioenergetics, respiration rates, membrane potential, mtROS, and ions, permeabilized cells were previously used to elucidate the CRC using different Ca^2+^-sensitive fluorescent probes [117,118]. As mentioned above, two biological detergents (digitonin and saponin) are primarily used to permeabilize cells; digitonin disrupts the plasmalemma by targeting lipid rafts, while saponins permeabilize it by selectively removing cholesterol from the membranes without affecting membrane proteins [6,34]. Importantly, permeabilization does not affect the essential subcellular organization and the structural and functional integrity of cellular organelles. We have recently shown that the mitochondrial CRC measured by the Ca^2+^-sensitive fluorescence probe Calcium Green-5N in saponin-permeabilized cardiomyocytes was significantly higher than in isolated mitochondria [118] (Figure 3). Furthermore, a comparative analysis of the permeabilization capacity of saponin and digitonin revealed that saponin-permeabilized cardiomyocytes exhibited a higher CRC than digitonin-permeabilized cells. This study, along with studies from other groups, suggests that analysis of the CRC in saponin-permeabilized intact cells has more advantages compared to the isolated mitochondria. Analysis of the CRC in permeabilized cells can be used in basic research as well as to diagnose different human diseases.

## 7. Mitochondrial Fluorescent Confocal Imaging

**Figure 4:**
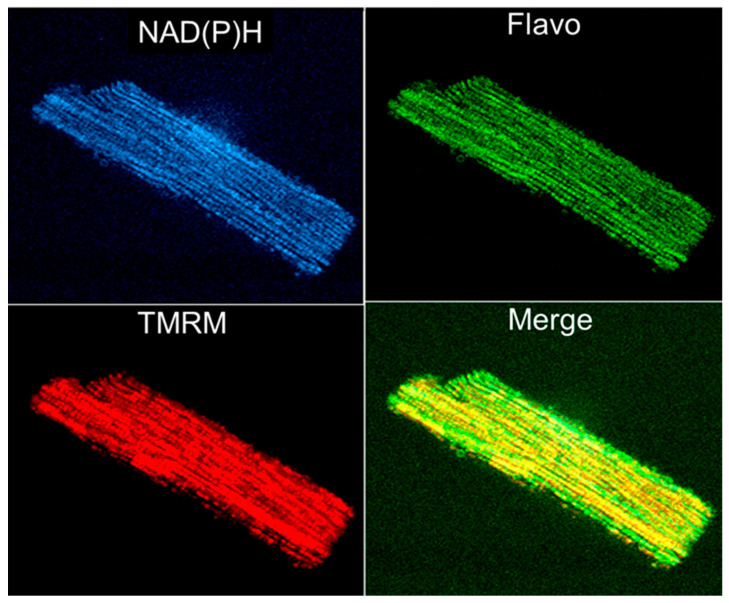
Simultaneous imaging of endogenous autofluorescence of mitochondrial NAD(P)H (two-photon excitation), flavoproteins (488 nm laser excitation), and membrane potential sensitive probe TMRM (see Figure 1 legend) in an adult rat single cardiomyocyte.

Rhodamine 123 was the first fluorescent dye used in flow cytometry [119] and in mitochondrial imaging. As it is specifically and potential-driven concentrated in mitochondria in living cells, rhodamine 123 was considered a useful probe for monitoring the abundance of mitochondria. However, due to the relatively low resolution, its application in imaging was limited. Applying techniques for autofluorescence and digital fluorescence imaging of mitochondrial NAD(P)H, endogenous flavoproteins, and specific mitochondrial fluorescent probes to permeabilized muscle fibers and cardiomyocytes (Figure 4) provided options for more precise imaging [38,39,42,63,65,87,120].

**Figure 5:**
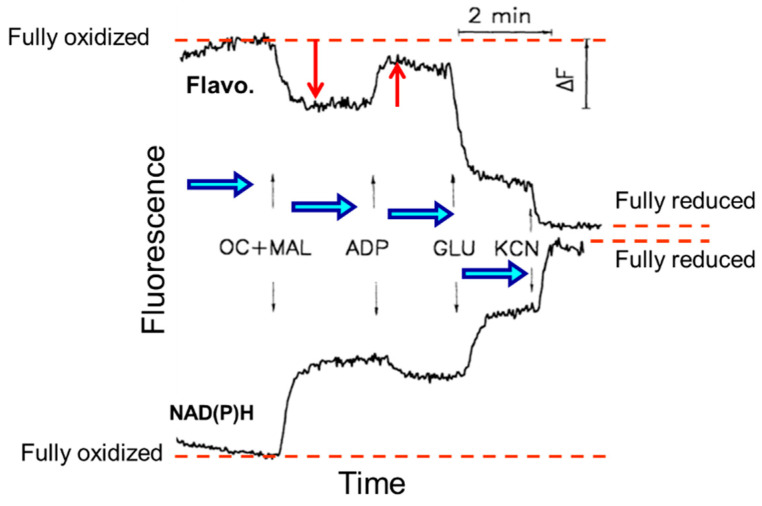
Quantitative measurements of flavoproteins (Flavo.) and NAD(P)H fluorescence and their responses to substrates additions of octanoylcarnitine and malate (OC + MAL), ADP, glutamate (GLU), and inhibitor potassium cyanide (KCN) from fully oxidized to a fully reduced state in permeabilized skeletal rat skeletal muscle (quadriceps femoris).

It is noteworthy that the imaging approach enables not only the observation of mitochondrial structure/morphology but also the ability to obtain information about the general mitochondrial properties and functions. Since mitochondrial flavoproteins are fluorescent in an oxidized state and NAD(P)H in a reduced state, it has been possible to continuously monitor mitochondrial redox states. The addition of mitochondrial substrates, ADP, or potassium cyanide resulted in strong changes in the NAD redox system. The intensities of fluorescent flavoproteins and NAD(P)H demonstrated inverse fluorescence signal behavior (Figure 5).

Importantly, flavoproteins and NADH fluorescence were fully co-localized with MitoTracker^TM^ Green FM, an established fluorescent marker for mitochondria. It has been shown that the ratio of the intensities of fluorescent flavoproteins and NAD(P)H is practically non-sensitive to any other types of fluorescence, thereby eliminating the possible side effects of artificial fluorescent probes and can therefore be used as a sensitive indicator of mitochondrial redox states. Using the imaging approach, the phenomenon of mitochondrial heterogeneity has been established. For example, a much higher oxidative state of subsarcolemmal as compared to intermyofibrillar mitochondria has been found [39]. The flavoprotein autofluorescence signals of these mitochondrial subpopulations were four times different. The identification of the membrane potential and mtROS also revealed the heterogeneity of pathologically altered mitochondria (e.g., after cold ischemia-reperfusion and transplantation of rat hearts) [121]. Mitochondrial imaging, therefore, permits the assessment of mitochondrial defects topology, which provides information about the molecular mechanisms of cardiac cold ischemia-reperfusion injury. Similarly, flavoprotein redox states and mitochondrial membrane potential heterogeneity have been demonstrated in intact cardiomyocytes under conditions of substrate (glucose) deprivation [40]. This included metabolic transients, well-coordinated redox transitions, and wave-like metabolic propagation within one cell and even between cells. The mechanism may involve some diffusible cytosolic messengers. Therefore, in addition to respirometry, fluorescence imaging approaches can be used for the analysis of functional mitochondria in permeabilized muscles and intact cardiac cells. Altogether, analysis of the mitochondrial structural organization, bioenergetics, and redox status using the aforementioned in situ techniques is useful for the precise estimation of the cardioprotective and anti-aging effects of newly developed mitochondria-targeted compounds [122,123,124,125,126,127]. Mitochondria-targeted antioxidants such as SS-31, MitoQ, XJB-5-131, SkQ, CoQ10, SOD mimetics, mitochondria-targeting glutathione (mitoGSH), and polyphenols, among others, could be examined in future studies.

## 8. Conclusions

Respirometry and imaging of mitochondria in intact or permeabilized cardiomyocytes and muscle fibers and tissues are reliable tools for the functional analysis of mitochondria in situ or in vivo, associated with preserved essential interactions with other intracellular systems. These approaches can be used in basic research as well as various clinically oriented studies where the minimization of sample sizes is an important advantage for analyzing mitochondria in human biopsies. In addition, imaging of endogenous fluorescent flavoproteins, NADH or mitochondria-specific fluorescent dyes, and genetically encoded fluorescent proteins can also be applied in mitochondrial studies. We reviewed methods that are applied to elucidate mitochondrial morphology, mitochondrial membrane potential, ion homeostasis, mitochondrial pH regulation, redox state transitions, mtROS production, and PTP opening/CRC in situ. Biochemical and functional characteristics of mitochondria obtained using in situ methods are markedly different from those obtained in isolated mitochondria in vitro. In summary, we reviewed currently available methods to study mitochondrial function, structure, and intracellular organization in situ in cardiomyocytes and skeletal muscle fibers.
